# Spatial navigation, aging and Alzheimer’s disease

**DOI:** 10.18632/aging.101634

**Published:** 2018-11-04

**Authors:** Jan Laczó, Martina Parizkova, Scott D. Moffat

**Affiliations:** 1 Memory Clinic, Department of Neurology, Charles University, 2nd Faculty of Medicine and Motol University Hospital, Prague, Czech Republic; 2 International Clinical Research Center, St. Anne’s University Hospital Brno, Brno, Czech Republic; 3 School of Psychology, Georgia Institute of Technology, Atlanta, GA 30313, USA

**Keywords:** aging, Alzheimer’s disease, spatial navigation, hippocampus, basal forebrain

Spatial navigation is a fundamental behavior of animals and humans and involves processes of planning a route and executing movements towards environmental goals. While there are many components to successful navigation, two frequently cited navigation strategies, egocentric (self-centered) and allocentric (world-centered), use different types of spatial reference frames to develop internal representations of surrounding environment. Egocentric navigation is a navigation strategy, where spatial information about locations and objects is encoded from the viewpoint of the navigator to form a self-centered spatial reference frame (self-to-object representations). Allocentric navigation is a navigation strategy, where locations and objects are encoded in relation to one another independently of the position of the navigator to form a world-centered spatial reference frame (object-to-object representations). Navigation is an inherently complex and multi-modal cognitive process and consequently, a large network of brain regions is recruited when navigating our environment. These include the medial temporal lobe, especially the hippocampus, entorhinal cortex and parahippocampal gyrus, the retrosplenial cortex and other regions of the parietal lobe and the prefrontal cortex. Some research indicates that the hippocampus and related medial temporal lobe structures play a prominent role in allocentric navigation and the precuneus and the caudate nucleus play a more prominent role in egocentric navigation [1].

Healthy aging is associated with mild functional and structural changes primarily in the prefrontal cortex and the medial temporal regions resulting in subtle and selective decline of executive functions, attention, working memory, episodic memory and spatial navigation. With respect to spatial navigation, older adults tend to show increasing preferences for egocentric strategies that could be the consequence of functional and structural changes in the hippocampus and related medial temporal lobe structures and may reflect the adoption of extrahippocampal navigation strategies [1,2].

Alzheimer’s disease (AD) is characterized by widespread neurodegeneration in the medial temporal lobe including the hippocampus, parietal lobe, prefrontal cortex and basal forebrain resulting in severe global cognitive decline, where impairment of episodic memory and spatial navigation is one of the most profound. Based on the severity of clinical impairment, the three stages of AD have been identified – (1) AD dementia, where severe global cognitive decline interferes with functional abilities; (2) mild cognitive impairment (MCI) due to AD, where selective modest cognitive decline does not interfere with functional abilities; and (3) preclinical AD that is characterized by the presence of neurodegenerative changes with no evident cognitive impairment on clinical assessment. Individuals with AD are frequently disoriented in unfamiliar environments and later in the course of the disease they may become lost in familiar places. Allocentric strategy is severely impaired in individuals with AD dementia and is also impaired in individuals with MCI due to AD and preclinical AD [3,4]. Egocentric strategy appears to be impaired in individuals with AD dementia and MCI due to AD but not in preclinical AD [3,4].

Functional and structural changes of the hippocampus and basal forebrain in AD are related to impairment of allocentric strategy [5]. Much less is known, however, about whether this dysfunction may lead to the recruitment of compensatory extrahippocampal navigation strategies, expressed as an increasing preference of the egocentric strategy, in the early clinical stages of AD (i.e. in individuals with amnestic MCI due to AD and mild AD dementia). To explore the differences in navigation strategy preference, its association with allocentric navigation performance and the role of hippocampal and basal forebrain volumes in this association, Parizkova et al. [6] used a virtual Y-maze strategy assessment task, a real-space human analogue of the Morris water maze task and magnetic resonance imaging brain volumetry in participants with mild AD dementia, amnestic MCI due to AD and cognitively normal older adults. The study showed that preference for the egocentric (i.e. extrahippocampal) strategy was more pronounced among participants in the early clinical stages of AD than in cognitively normal older adults and that this strategy preference increased with AD severity. The participants in the early clinical stages of AD had less accurate real-space allocentric navigation performance and the preference for the egocentric strategy in participants with aMCI due to AD was associated with less accurate allocentric navigation on an independent real-space assessment. To determine the nature of the association between navigation strategy preference and allocentric navigation performance, Parizkova et al. investigated the potential role of hippocampal and basal forebrain volumes in mediation of this interrelation. The results showed that lower hippocampal and basal forebrain volumes were associated with less accurate real-space allocentric navigation and explained up to 25% of the association between navigation strategy preference and real-space allocentric navigation performance in participants with aMCI due to AD. In addition, right hippocampal and basal forebrain volumes acted synergistically such that low right hippocampal volume further increased the tendency of participants with low basal forebrain volume to use egocentric rather than allocentric strategy. In summary, these data indicated that AD-related neurodegenerative changes in the hippocampus and basal forebrain may lead to the less effective allocentric navigation processing and reduced reliance on the allocentric strategy. This in turn may lead to compensatory recruitment of egocentric (i.e. extrahippocampal) strategies in the early clinical stages of AD.

Spatial navigation deficits may be a promising cognitive marker for identifying individuals with incipient AD. Given that spatial navigation is distinguishable from other cognitive functions, its assessment along with examination of other major cognitive functions could be highly beneficial when characterizing the cognitive profile of healthy older adults and individuals with AD [7].
